# A mathematical model for mechanical activation and compound action potential generation by the utricle in response to sound and vibration

**DOI:** 10.3389/fneur.2023.1109506

**Published:** 2023-03-27

**Authors:** Christopher J. Pastras, Nastaran Gholami, Skyler Jennings, Hong Zhu, Wu Zhou, Daniel J. Brown, Ian S. Curthoys, Richard D. Rabbitt

**Affiliations:** ^1^Faculty of Science and Engineering, School of Engineering, Macquarie University, Sydney, NSW, Australia; ^2^Biomedical Engineering, University of Utah, Salt Lake City, UT, United States; ^3^Communication Sciences and Neuroscience Program, University of Utah, Salt Lake City, UT, United States; ^4^University of Mississippi Medical Center, Jackson, MS, United States; ^5^School of Pharmacy and Biomedical Sciences, Curtin University, Bentley, WA, Australia; ^6^Vestibular Research Laboratory, School of Psychology, The University of Sydney, Sydney, NSW, Australia; ^7^Otolaryngology and Neuroscience Program, University of Utah, Salt Lake City, UT, United States

**Keywords:** vestibular, vestibular evoked miogenic potentials, vestibular short-latency evoked potential, action potential timing, biomechanics

## Abstract

**Introduction:**

Calyx bearing vestibular afferent neurons innervating type I hair cells in the striolar region of the utricle are exquisitely sensitive to auditory-frequency air conducted sound (ACS) and bone conducted vibration (BCV). Here, we present experimental data and a mathematical model of utricular mechanics and vestibular compound action potential generation (vCAP) in response to clinically relevant levels of ACS and BCV. Vibration of the otoconial layer relative to the sensory epithelium was simulated using a Newtonian two-degree-of-freedom spring-mass-damper system, action potential timing was simulated using an empirical model, and vCAPs were simulated by convolving responses of the population of sensitive neurons with an empirical extracellular voltage kernel. The model was validated by comparison to macular vibration and vCAPs recorded in the guinea pig, in vivo.

**Results:**

Transient stimuli evoked short-latency vCAPs that scaled in magnitude and timing with hair bundle mechanical shear rate for both ACS and BCV. For pulse BCV stimuli with durations <0.8 ms, the vCAP magnitude increased in proportion to temporal bone acceleration, but for pulse durations >0.9 ms the magnitude increased in proportion to temporal bone jerk. Once validated using ACS and BCV data, the model was applied to predict blast-induced hair bundle shear, with results predicting acute mechanical damage to bundles immediately upon exposure.

**Discussion:**

Results demonstrate the switch from linear acceleration to linear jerk as the adequate stimulus arises entirely from mechanical factors controlling the dynamics of sensory hair bundle deflection. The model describes the switch in terms of the mechanical natural frequencies of vibration, which vary between species based on morphology and mechanical factors.

## Introduction

Auditory frequency ACS and BCV are commonly used to activate vestibular otolith organs in the inner ear for basic science applications and as part of the neuro-otology clinical test battery. Utricular and saccular afferent neurons with irregularly spaced inter-spike intervals are the most sensitive to sound and vibration (1–3), and for sinusoidal stimuli fire action potentials at a precise phase in the stimulus cycle. Transient pulse or click stimuli evoke synchronized action potential firing in these sensitive neurons, resulting in detectable whole-nerve vestibular compound action potentials (vCAPs), similar to extracellular field potentials first observed in peripheral nerves a century ago (4). Vestibular short-latency evoked potentials (VsEPs) are specific vCAPs commonly measured for vestibular phenotyping in animal models via subcutaneous electrodes in response to whole-skull nasal-occipital vibration (5, 6). The function of the vestibular system can also be monitored through compensatory reflex responses of neural-muscular circuits including the vestibular-ocular, −spinal, and -colic systems (7). In humans, vestibular evoked myogenic potentials (VEMPs) in ocular and cervical muscle groups are routinely measured in the clinic in response to sound or vibration to assess the function of the utricle and saccule, respectively, (8–11). Although ACS and BCV stimuli are commonly used to activate otolith organs, precisely how these stimuli deflect hair bundles, activate mechano-electrical transduction (MET) currents, and evoke synchronized action potentials in vestibular otolith afferent neurons remains unknown.

In the traditional view, the otolith organs are gravito-inertial sensors responsible for detecting orientation of the head relative to gravity and low-frequency linear acceleration (7). Consistent with this, most mechanical models of otolith organs have focused on slowly changing inertial forces and treat the utricle as a one degree-of-freedom (1-DOF) spring-mass-damper system forced by gravity or classical base-support vibration (12, 13). With appropriate parameters, 1-DOF mechanical models capture the low-pass nature of otoconial vibration in response to gravito-inertial acceleration (14, 15), but they fail to address activation by ACS and fail to describe vibration of the epithelium relative to the skull. In the present report we introduce another degree of freedom to allow the membranous labyrinth to vibrate relative to the temporal bone, and allow the otoconial layer to vibrate relative to the macular epithelium, with the difference between the two deflecting hair bundles and gating mechano-electrical transduction (MET) channels. We combined this 2-DOF mechanical model with an empirical integrate-and-fire to simulate synchronized vestibular afferent action potential firing and resulting whole-nerve vestibular compound action potentials (vCAPs). To validate the model, we compared theoretical predictions in response to BCV and ACS stimuli to macular vibrations measured using laser doppler vibrometry, and to vCAPs recorded near the vestibular nerve in guinea pigs, in vivo. Once validated, the model was applied to elucidate the origins of utricular mechanical activation and synchronized action potentials in response to clinically relevant ACS and BCV stimuli, and in response to damaging acoustic blast exposure.

## Materials and methods

### Experiments

**Figure 1:**
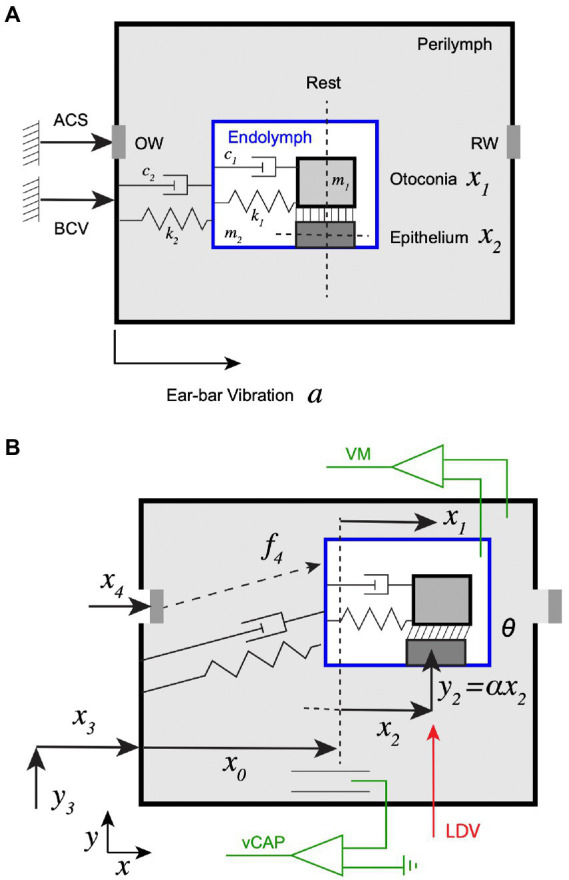
Experimental set up and schematic of the mechanical model. **(A)** Two different mechanical stimuli were used: (1) bone conducted vibration (BCV), quantified experimentally by measuring acceleration ″a″ of the ear-bar fixture, (2) air conducted sound (ACS) quantified experimentally by measuring stapes velocity ${}^{“}v={\frac{dx_{4}}{dt}}^{“}$ . BCV was modeled as vibrating the entire perilymphatic space, while ACS was modeled as vibrating the oval window (OW) and round window (RW) simultaneously. The membranous labyrinth was treated as a lumped mass tethered to the temporal bone through viscoelastic elements, and the utricular otoconial layer was treated as a second lumped mass tethered to the utricular sensory epithelium by viscoelastic elements. **(B)** Mechanical displacements of the masses and the round window are denoted $\left(x_{n},y_{n}\right)$ and angular shear between the otoconial layer and the surface of the epithelium is denoted θ . Vestibular compound action potentials (vCAP) were recorded in perilymph relative to ground, vestibular microphonics (VM) were recorded across the membranous labyrinth and vibration of the epithelium was recorded in the y direction with an LDV system.

Data reproduced in the present study for model development and parameter estimation represent a subset of a larger study by Pastras et al. (16). All procedures were approved by the University of Sydney Animal Ethics Committee (Protocol# 2019/1533). Experimental methods are detailed elsewhere (16–18). Briefly, vCAPs, macular vibration, stapes vibration, and ear-bar (temporal bone) vibration were recorded in healthy anaesthetized guinea pigs (Cavia porcellus) as shown schematically in Figure 1. vCAPs were recorded relative to a reference electrode in the neck musculature using a Ag/AgCl electrode in the bony facial nerve canal, near the superior branch of the vestibular nerve. Linear acceleration was measured at the ear-bar fixture, adjacent to the skull, using a 3-axis accelerometer.

For measurements of utricular macular and stapes vibration, a single-point Laser Doppler Vibrometer (LDV) (Type 8,338, Brüel & Kjær, Denmark) was used with high-velocity capability (max. 500 mm/s) and wide frequency bandwidth, up to 22 kHz. The system resolution was ≤0.02 μm/s/√Hz, and the dynamic range was >90 dB over its full bandwidth. The bony labyrinth was opened to allow optical access for LDV recordings. To enhance signal strength, 20 μm diameter reflective glass microbeads (Refractive index >1.93; Cospheric, CA, United States) were placed onto the macular epithelium and stapes footplate, under the guidance of a surgical microscope using a ventral surface approach. The laser beam (628 nm, red) was directed onto the microbead targets via an optical mirror (Thorlabs, United States), which was adjustable in 3D. The level of fluid was managed by tissue wicks to avoid microbead immersion in perilymph and artifacts via fluid surface motion introducing frequency shifts in the LDV signal (19). For ACS stimulation, the tympanum and ossicular chain were left intact, and the fluid level in the bony labyrinth at the surgical opening was maintained to ensure direct fluid coupling between the stapes footplate and macula.

### Mechanics

We developed a simple 2-DOF model to approximate mechanical excitation of the utricle by ACS and/or BCV. A schematic of the mechanical system at rest is provided in Figure 1A and in a deformed configuration during stimulation in Figure 1B. BCV was modelled as vibrating the temporal bone with acceleration $a_{b}=\frac{d^{2}x_{3}}{dt^{2}}$ relative to inertial ground, and ACS was modelled by an inertial force applied to the membranous labyrinth proportional to stapes acceleration $a_{s}=\frac{d^{2}x_{3}}{dt^{2}}$ . At rest, the sensory epithelium was locatedat distance $x_{0}$ from the bony capsule. Displacement of the sensory epithelium relative to the bone was defined as $x_{2}\left(t\right)$ , displacement of the otoconial layer relative to the bone was defined as $x_{1}\left(t\right)$ , and shear angle between the layers as θ(t) . Shear is the key mechanical variable because it is directly related to hair bundle deflection while displacements of the individual layers are not (e.g., if $x_{1}=x_{2}$ the hair bundle deflection is zero). The otoconial layer was modelled as a single mass $m_{1}$ tethered to the sensory epithelium by an elastic element of stiffness $k_{1}$ and a viscous element with damping coefficient $c_{1}$ . The utricular membranous labyrinth and epithelial layer were assumed to be tethered to the temporal bone by stiffness $k_{2}$ and damping coefficient $c_{2}$ , with inertia modelled with a single effective mass $m_{2}$ . For small vibrational stimuli, we assumed the membranous labyrinth moved in a straight line with x,y and z components linearly related to each other. Specifically, for a vibrational stimulus in $\hat{i}$ ( x ) direction the position of the epithelium was assumed to have the form $\vec{u}=\left(x_{3}+x_{0}+x_{2}\right)\hat{i}+\gamma x_{2}\hat{j}+\beta x_{2}\hat{k}$ , where γ and β are constants. With this simplification, Newton’s second law in the $\hat{i}$ direction gives the equation of motion for vibration of the otoconial layer tangent to the epithelium as


(1) $$\frac{d^{2}x_{1}}{dt^{2}}+2\zeta_{1}\omega_{1}\frac{dx_{1}}{dt}+\omega_{1}^{2}x_{1}=f_{1},$$

where the natural frequency is $\omega_{1}^{2}=\frac{k_{1}}{m_{1}}$ and the nondimensional damping coefficient is $\zeta_{1}=\frac{c_{1}}{2\;m_{1}\omega_{1}}$ . The forcing term on the right-hand side is:


(2) $$f_{1}=-\beta_{1}\frac{d^{2}x_{3}}{dt^{2}}+2\zeta_{1}\omega_{1}\frac{dx_{2}}{dt}+\omega_{1}^{2}x_{2}.$$

The equation of motion for vibration of the epithelium is:


(3) $$\frac{d^{2}x_{2}}{dt^{2}}+2\zeta_{2}\omega_{2}\frac{dx_{2}}{dt}+\omega_{2}^{2}x_{2}=f_{2}$$

where the natural frequency is $\omega_{2}^{2}=\frac{k_{2}}{m_{2}}$ and the otoconial nondimensional damping coefficient is $\zeta_{2}=\frac{c_{1}+c_{2}}{2\;m_{2}\omega_{2}}$ . The force on the right-hand side of Eq. 3 is:


(4) $$f_{2}=-\beta_{2}\frac{d^{2}x_{3}}{dt^{2}}-\alpha \frac{d^{2}x_{4}}{dt^{2}}+2\zeta_{1}\omega_{1}r\frac{dx_{1}}{dt}+\omega_{1}^{2}rx_{1}$$

where r is the ratio of the otoconial mass to the effective mass of the endolymph-filled labyrinth (including the epithelium) is $r=\frac{m_{1}}{m_{2}}$ . Since the total mass of the endolymph is much greater than the otoconial mass, the ratio r≪1 . The term $\alpha \frac{d^{2}x_{4}}{dt^{2}}$ is the inertial force exerted by the perilymph on the labyrinth caused by acceleration of the stapes. Neglecting terms multiplied by r , we approximate $f_{2}$ using:


(5) $$f_{2}\approx -\beta_{2}\frac{d^{2}x_{3}}{dt^{2}}-\alpha \frac{d^{2}x_{4}}{dt^{2}}.$$

The first term, $\beta_{2}\frac{d^{2}x_{3}}{dt^{2}}$ , arises from BCV and the second term, $\alpha \frac{d^{2}x_{4}}{dt^{2}}$ , arises from ACS induced stapes vibration. When the bony labyrinth is intact, the factors $\beta_{1}$ and $\beta_{2}$ account for the acceleration induced pressure in the fluids, which reduces the effective mass in analogy to buoyancy (e.g., $\beta_{1}=1-\rho /\rho_{1}$ , where ρ is the endolymph density and $\rho_{1}$ is the otoconial layer density) (20, 21). Since the bony labyrinth was open in the present study, pressure in the endolymph and perilymph was assumed uniform which reduces both factors to $\beta_{1}=\beta_{2}=1.$ For BCV simulations, the stimulus was determined from ear-bar accelerometer data $a_{b}=\frac{d^{2}x_{3}}{dt^{2}}$ , and for ACS simulations the stimulus was determined from the derivative of stapes velocity $v=\frac{dx_{4}}{dt}$ measured using LDV.

We solved Eq. 3 and Eq. 1 in the time domain using the following convolution integral:


(6) $$x_{n}\left(t\right)=\overset{t}{\underset{0}{\int }}G_{n}\left(t,t^{\prime }\right)f_{n}\;\left(t^{\prime }\right)dt^{\prime }.$$

For $\zeta_{n}<1$ , the Green’s function is:


(7) $$\begin{array}{l} G\left(t,t^{\prime }\right)=H\left(t-t^{\prime }\right)\left(\omega_{n}^{2}-{\left(2\zeta_{n}\omega_{n}\right)}^{2}\right)\\ \sin\left(\sqrt{\omega_{n}^{2}-{\left(2\zeta_{n}\omega_{n}\right)}^{2}}\left(t-t^{\prime }\right)\right)e^{-2\zeta_{n}\omega_{n}}, \end{array}$$

where H is the unit step function. Eq. 3 was first solved to approximate vibration of the epithelial layer $x_{2}$ (neglecting r ), which was then substituted into Eq. 1 to solve for vibration of the otoconial layer $x_{1}$ . Angular shear acting on hair bundles was approximated as


(8) $$\vartheta \left(t\right)=Atan\left(\frac{x_{1}-x_{2}}{h}\right)$$

where h is the distance from the epithelial surface to the otoconial layer.

### Mechanical parameter estimation

There are five parameters in the mechanical model that need to be estimated from experimental data: two natural frequencies $\omega_{1},\omega_{2}$ (rad-s^−1^), two nondimensional damping coefficients $\zeta_{1},\zeta_{2}$ and an inertial coefficient α governing the magnitude of the inertial force acting on the labyrinth due to stapes acceleration. Specifically, the natural frequencies and the damping coefficients in Table 1 were estimated from the temporal waveform of the macular vibration measured in response to applied BCV stimuli, and the inertial coefficient was found from the magnitude of macular vibration measured in response to applied ACS stimuli.

**Table 1:** Model parameters.

α	0.3	Nondimensional
$g_{0}$	0	Nondimensional
$g_{1}$	0	Nondimensional
$g_{2}$	4*10^3^	Nondimensional
r	0	Nondimensional
$T_{r}$	0.003	s
$T_{d}$	0.0003	s
$T_{e}$	0.001	s
τ	0.01	s
$\omega_{1}$	520*2*π	rad-s^−1^
$\omega_{2}$	1240*2*π	rad-s^−1^
$\zeta_{1}$	0.3	Nondimensional
$\zeta_{2}$	0.9	Nondimensional

### Short latency compound action potentials

We used an empirical integrate-and-fire (IAF) model with an absolute refractory period to simulate action potential times. The IAF model was driven by synaptic current via hair cell depolarization. The most sensitive phase locking utricular afferents make calyceal synaptic contacts with type 1 hair cells in the striola. The unique ultrafast nonquantal component of synaptic transmission at the calyx (22–25) is likely key to short latency evoked action potentials (26), but the present approach is heuristic and makes no attempt to capture the biophysics. For simplicity, we assumed the mechano-electrical transduction current was driven by hair bundle shear ϑ , and that the mechanical stimulus was slower than the type-1 hair cell membrane time constant. With these simplifications, the perturbation in hair cell voltage for small stimuli becomes proportional to the bundle shear v∝ϑ . Ionic currents in the afferent neuron were assumed to be sensitive to voltage as well as the rate of change in voltage. With this we assumed the net depolarizing current exciting the sensitive afferent neurons was proportional to ϑ and $\frac{d\vartheta }{dt}$ . A simple IAF equation was used to find the mean spike time relative to the previous mean spike time for the entire population of sensitive neurons, with distributions around each mean time used to simulate the stimulus evoked histogram for the population. Considering only short-latency synchronized responses of calyx bearing afferents, we related the mean probability $p_{n}$ of evoking a spike in time bin ${}^{"}n^{\prime \prime }$ to the probability at the previous time $p_{n-1}$ and the input currents by Euler integration:


(9) $$p_{n}=p_{n-1}+\Delta t\left(-\frac{p_{n-1}}{\tau }+g_{0}\frac{1}{\tau }+g_{1}\frac{\vartheta_{n}}{\tau }+g_{2}\frac{d\vartheta_{n}}{dt}\right),$$

where ∆*t* is the time step, τ is the mean afferent integration time constant, $g_{0}$ is a pacemaker gain for neurons with regular firing, $g_{1}$ is a gain that sets sensitivity to changes in bundle shear, and $g_{2}$ is a gain that sets sensitivity to the rate of change in bundle shear. The rate of change sensitivity ( $g_{2}$ ) in this empirical approach arises primarily from an adaptation process interposed between the MET and the action potential spike train characteristic of afferent neurons sensitive to high frequency stimuli (27, 28). The simulation starts at time $t_{0}=0$ and integrates forward using the shear ϑ in Eq. 8 until $p_{m}=1$ , which defines the mean time $T_{m}$ of stimulus evoked action potentials. After finding the mean action potential time $T_{m}$ , the probability is set to zero for an absolute refractory time $T_{R}$ and integration of Eq. 9 is resumed starting at time $t=T_{m}+T_{R}$ .

We assumed the variability in gains and integration times across the population of neurons to be Gaussian, and that parameters in Eq. 9 yield the mean time $T_{m}$ of action potential generation. We consider only the short latency vCAP, and ignore the excitatory-inhibitory nonlinearity. The number of afferent fibers recruited around mean time $T_{m}$ is $R_{m}=R\left(\dot{\vartheta }\right)$ where R is a saturating function of bundle shear rate $\dot{\vartheta }$ . Specifically, for calyx bearing afferent neurons sensitive to transient stimuli we used a saturating nonlinearity:


(10) $$R=N\left(1-\exp\left(-\dot{\vartheta }/{\dot{\vartheta }}_{0}\;\right)\right),$$

where N is the maximum number of units recruited per time bin, and ${\dot{\vartheta }}_{0}$ is the shear rate governing saturation to the maximum number of excitable units. The stimulus-evoked spike histogram around mean time $T_{m}$ gives the post-stimulus histogram describing the number of action potentials:


(11) $$P\left(t\right)=\overset{M}{\underset{m=1}{\sum }}\frac{R_{m}}{\sigma_{m}\sqrt{2}\pi }\exp\left(-\frac{1}{2}{\left(\frac{t-T_{m}}{\sigma_{m}}\right)}^{2}\right),$$

where the square root of the variance, σ , is assumed inversely proportional to R . Using a form similar to Chertoff et al. (29) for auditory nerve CAPs, the extracellular voltage generated by a single action potential (i.e., “unit response”) at time $t^{\prime }$ is written in the form:


(12) $$\begin{array}{l} u\left(t,t^{\prime }\right)=A\;H\left(t-t^{\prime }\right)\;\sin\left(2\pi \left(t-t^{\prime }\right)/T_{e}\right)\\ \left\{\begin{array}{cc} 1, & \left(t-t^{\prime }\right)<T_{e}\\ e^{-\left(t-t^{\prime }-T_{e}\right)/\tau_{e}}, & \left(t-t^{\prime }\right)\geq T_{e} \end{array}\right. \end{array}$$

where $\tau_{e}$ is the exponential decay time constant and $T_{e}$ is the period of the extracellular voltage waveform. Convolution with the spike probability (30) provides the vestibular compound action potential *vCAP*


(13) $$vCAP\left(t\right)=\overset{t}{\underset{-\infty }{\int }}P\left(t^{\prime }\right)u\left(t,t^{\prime }\right)dt^{\prime }.$$

### Neural parameter estimation

Equations 9–12 were selected heuristically with parameters in Table 1 estimated to fit the temporal waveform and timing of measured vCAP responses to BCV. The amplitude period, and decay time of the unitary waveform (Eq. 12: $A,T_{e},\tau_{e}$ ) were assumed constant and estimated to match the experimentally measured vCAP waveforms. Synchronized action potentials were assumed to arise from calyx bearing afferents sensitive primarily to the rate of change in bundle shear (Eq. 9: *g*_0_ ≈ 0, *g*_1_ ≈ 0). The gain $g_{2}$ in Eq. 9 determines the latency of synchronized action potentials, and was estimated from the latency of the vCAP responses. Saturation of the vCAP response as the mechanical stimulus was increased was used to estimate the parameter ${\dot{\vartheta }}_{0}$ (Eq. 10), which was also used to estimate $\sigma_{m}=20/R$ (Eq. 11). Finally, the product A⋅N appearing implicitly in Eq. 13 (from Eqs. 10–12) was adjusted so the simulated vCAP magnitude matched the measured magnitude. All data and simulations in the present report address short-latency responses generated by the first action potential evoked in each neuron, and hence the absolute refractory period $T_{R}$ was not relevant. All neural parameters were estimated using BCV data, and the exact same parameters were used for ACS simulations without modification.

## Results

### Simulated phase locking

**Figure 2:**
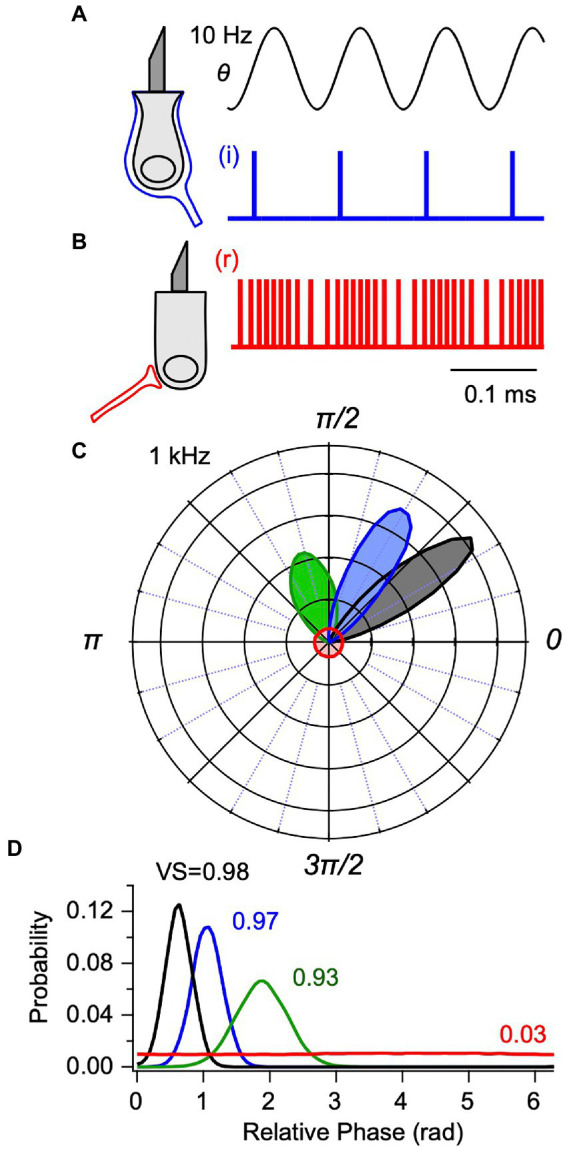
Integrate-and-fire (IAF) model for utricular afferent action potential generation. Simulated spike times are shown for: **(A)** a phase-locking afferent contacting type I hair cells with calyx [**(C)** blue, i-irregular] synaptic terminals and **(B)** a non-phase-locking afferent contacting type II hair cells with bouton [**(B)** red, r-regular] synaptic terminals (from Eq. 9). **(A,B)** Responses are shown for 10 Hz sinusoidal shear $\theta =\theta_{0}\sin\left(\omega t\right)$ generated by BCV. Phase-locking afferents fire action potentials at a precise phase relative to the sinusoidal stimulus, illustrated for one example neuron in response to 1,000 Hz BCV in **(C,D)** as polar and cartesian probability density functions, or phase histograms (Eq. 11). For the IAF model, the vector strength (VS) increases and the mean latency decreases as the stimulus strength increases (black: *VS* = 0.98, blue: 0.97, green: 0.93). For comparison, the phase histogram of regularly discharging unit that does not phase-lock to the 1,000 Hz stimulus is also shown (red).

Vestibular CAPs are generated by synchronized stimulus-evoked action potentials across a population of neurons. The degree of synchronization in the present IAF model arises from the gains ( $g_{0}$ , $g_{1}$ , $g_{2}$ ), and the variance in spike timing across the population arises from the parameter σ . To illustrate synchronization in the present model, Figures 2A,B show representative spike trains for an IAF simulated phase-locked neuron (i, blue, $g_{0}=0,g_{1}=0,g_{2}=4x10^{3}$ ) and for a simulated non-phase-locked neuron (r, red, $g_{0}=2.2,g_{1}=2x10^{6},g_{2}=0$ ) in response to BCV at 10 Hz. Action potential timing in the phase-locked example (A, i, blue) occurs at a phase closely aligned with the peak hair bundle shear rate ( $\dot{\vartheta }$ ). In contrast, action potential timing in the second example (B, r, red) modulates around a background discharge rate of ~95 spk-s^−1^ and, on average, timing of individual spikes has no relationship to phase of the stimulus. The difference in action potential timing is more clearly illustrated by the vector strength (VS, (31)) and the spike timing, shown as polar and linear phase histograms in Figures 2C,D for a 1,000 Hz BCV stimulus. The flat phase histograms with a vector strength of 0.03 were generated from the regularly discharging neuron (Figure 2B, red), while the three Gaussian curves of vector strength 0.93–0.98 were generated from the phase-locking neuron (Figure 2A, blue). In all simulations, Eq. 9 provided mean firing time while Eq. 10–11 provided the population distribution around the mean. Increasing the strength of the 1,000 Hz BCV stimulus increased the vector strength of the phase-locked action potentials from 0.93 (Figures 2C,D, green) to a saturated value of 0.98 (Figures 2C,D, black). Neurons that phase lock to sinusoidal stimuli synchronize to the onset of transient stimuli with an integration delay, and are responsible for short-latency vCAPs.

### Bone conducted vibration: Theory versus experiment

**Figure 3:**
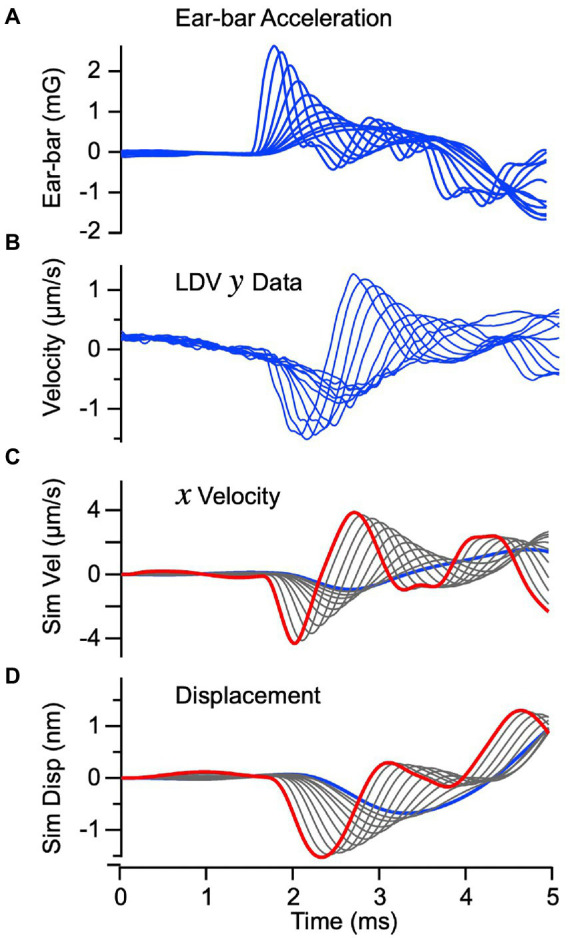
Vibration of the macula for BCV: Theory vs. Experiment. **(A)** Input acceleration of the ear-bar fixture “*a*” measured experimentally in the guinea pig (blue). Individual curves show responses for different voltage magnitudes and rise-times driving the vibrational stimulator. **(B)** Velocity of the epithelium measured by LDV in the “*y*” direction. **(C)** Simulated velocity of the epithelium in the “*x*” direction in response to temporal bone acceleration. The simulated velocity **(C)** closely follows the macula velocity measured in the “*y*” direction when scaled by the geometrical fator α≈0.3 . **(D)** Simulated displacement of the sensory epithelium in the tangential “*x*” direction.

To validate the mechanical part of the model we compared velocity of the epithelial layer (macula) predicted by the model to the velocity measured by LDV during BCV, in vivo. Figures 3A,B show the experimentally measured ear-bar (temporal bone) acceleration and macular velocity, with the series of curves corresponding to increasing stimulus strengths (16). The velocity was measured from the basal surface of the macular epithelium, opposite from the otoconial layer. The ear-bar acceleration (Figure 3A) was used as the input to the mathematical model to predict the macular velocity in Figure 3C and displacement in Figure 3D. The high temporal correspondence between the predicted x velocity (Figure 3C) and the experimentally measured y velocity (Figure 3B) confirms the model provides a reasonable description of the dynamics. If the macula vibrates along a straight line, as assumed in the model, the y velocity measured in panel B should be proportional to the x velocity predicted by the model in panel C, which is the case with γ ~0.3.

**Figure 4:**
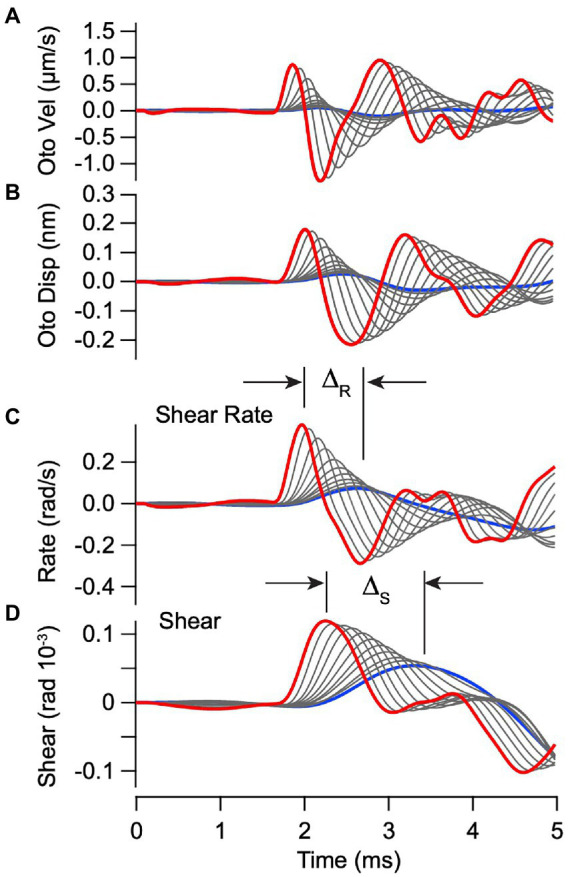
Simulated vibration of the otoconial layer for BCV. Individual curves use the same parameters and stimuli as Figure 3. **(A,B)** Otoconial layer velocity and displacement relative to the temporal bone. **(C,D)** Angular shear rate (radians/s) and shear (rad) between the otoconial layer and the epithelial surface (macula), which reflects angular deflection and the rate of angular deflection of the hair bundles. Δ is the delay between peak shear rate **(C)** and peak shear **(D)** in response to the highest vs. the lowest strength BCV stimuli.

The mechanical model also predicts velocity and displacement of the otoconial layer as shown in Figures 4A,B, driven by the ear-bar acceleration in Figure 3A. Displacement of the epithelial layer was subtracted from the otoconial layer to find the mechanical shear rate ( $\dot{\vartheta }$ , Figure 4C) and shear ( ϑ , Eq. 8, Figure 4D) acting on hair bundles. Previous single unit recordings show that vestibular afferent responses depend on both ϑ and $\dot{\vartheta }$ (27, 28), with $\dot{\vartheta }$ dominating for neurons that respond to high frequency transient stimuli. We therefore expected vCAPs evoked by BCV to be driven primarily by $\dot{\vartheta }$ ( $g_{2}$ in Eq. 9). One clear difference between the predicted bundle shear rate (Figure 4C) and shear (Figure 4D) is the latency between the highest stimulus strength (red) and the lowest stimulus strength (blue). As shown below, the stimulus level dependent timing of vCAPs is consistent with the latency of the bundle shear rate (Figure 4C, ∆_R_) vs. shear (Figure 4D, ∆_S_).

**Figure 5:**
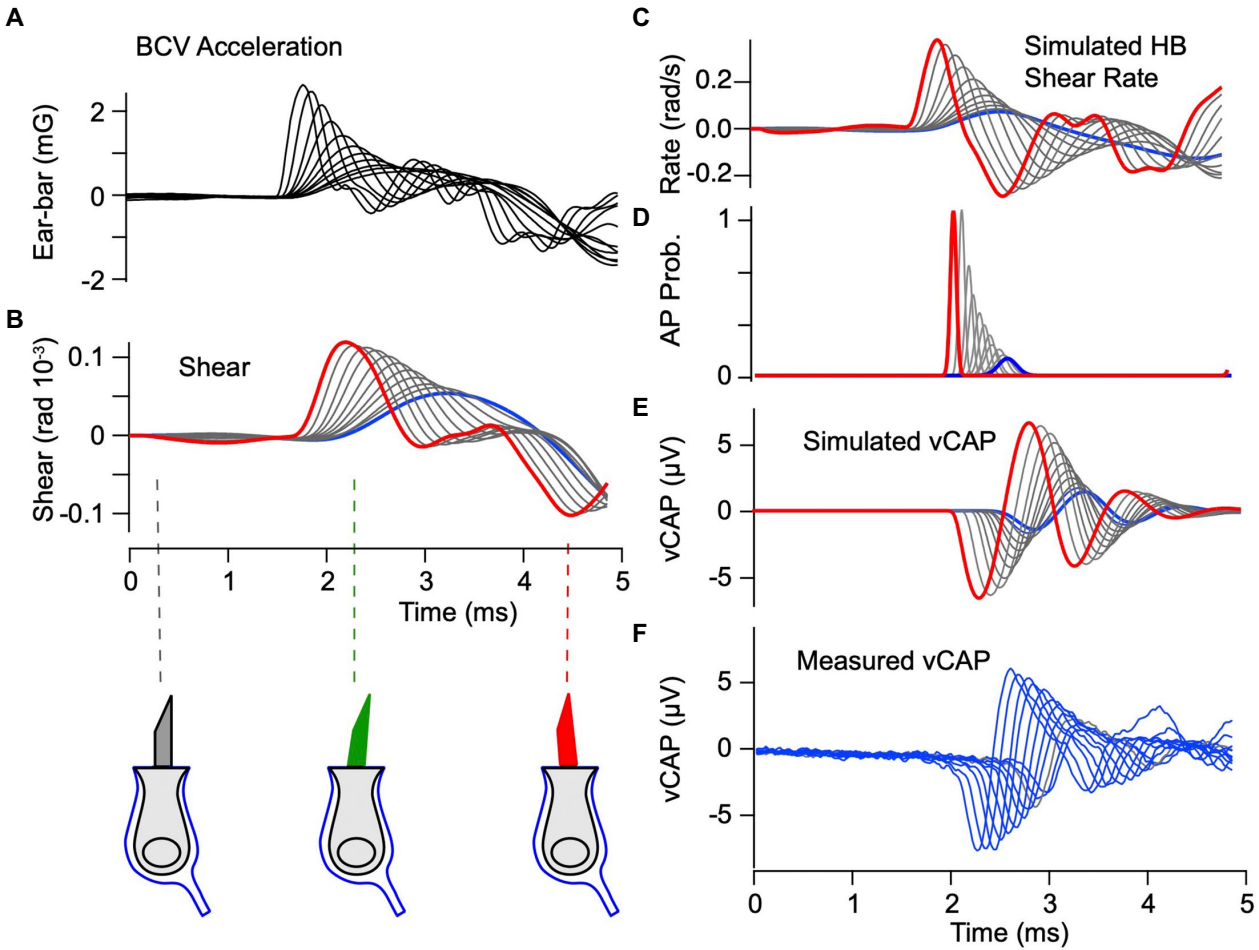
vCAP evoked by BCV: Theory vs. Experiment. **(A)** Experimentally measured ear-bar acceleration used as the input to the simulations. **(B)** Simulated shear driving hair bundles, which is predicted to have a peak magnitude of ~10^−4^ radians. Red curves indicate the highest strength stimulus and blue curves indicate the lowest strength. Direction and magnitude of hair bundle deflection is indicated below panel **B**. **(C)** Shear rate (time derivative of shear in panel **B**) driving the IAF model for phase locking afferent neurons. **(D)** Normalized probability of evoking an action potential as a function of time, where the magnitude corresponds to the number of units recruited, while the mean and width correspond to the most probable time and the variance. **(E)** Vestibular compound action potentials (vCAPs) simulated by convolving action potential probability with the field potential kernel (Eq. 12). **(F)** vCAPs measured in the anaesthetized guinea pig during the BCV stimuli.

**Figure 6:**
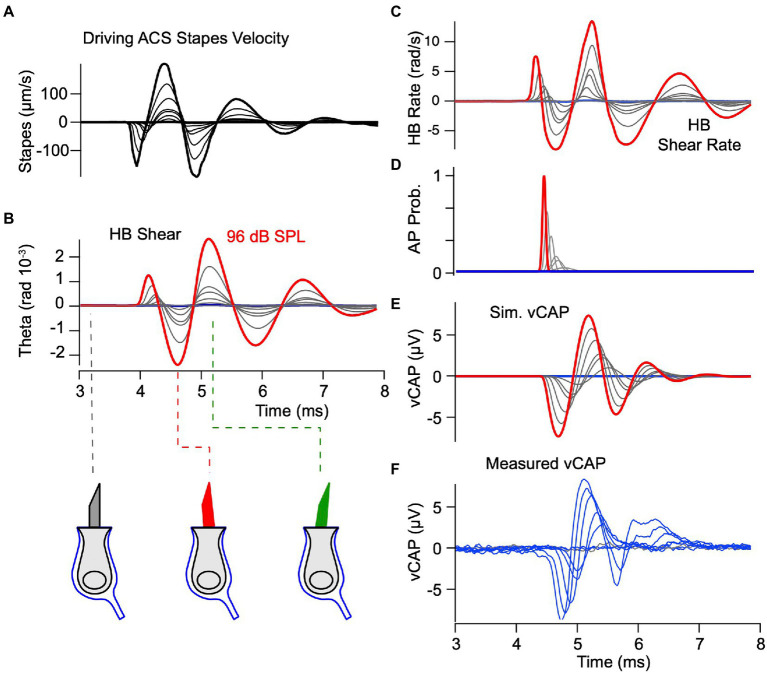
vCAP evoked by ACS. Theory vs. Experiment. Model parameters are exactly the same as previous figures, and the format is the same as Figure 5. **(A)** Experimentally measured vibration of the stapes used as the input to the simulations. **(B,C)** Simulated shear and shear rate activating hair bundles. **(D)** Probability of evoking a spike as a function of time. **(E,F)** Simulated and experimentally measured vCAPs.

To examine the origin of BCV evoked vCAPs, we compared model simulations to experimental data. The experimentally measured acceleration (Figure 5A) was used in the model as the stimulus driving the system. Predicted hair bundle shear (rad) and shear rates (rad-s^−1^) are shown in Figures 5B,C. The maximum shear magnitude was ~0.1×10^−3^ radians (Figure 5B), which corresponds to ~1.5 nm displacement at the bundle tip. The corresponding maximum shear rate had a magnitude of ~0.3 rad-s^−1^. As expected from the mechanical equations, there was no delay between the onset of the acceleration stimulus and the onset of the mechanical response, but mechanical rise-time of the shear rate plays a role in action potential timing as discussed below. The shear rate was used to drive action potential generation using Eq. 9 (note: $g_{0}=g_{1}=0$ in the present simulations), and post-stimulus histograms (Figure 5D) were found from Eqs. 10–11. Due to the absolute refractory period, each responding neuron fired only one action potential during the short 3.5 ms time period following onset of hair bundle motion. Differences in the Gaussian distributions (Figure 5D) arise from how fast action potentials are generated (latency), how many neurons are recruited (area under curve) and how precise synchronization is across the population (variance). Sensitivity of the vCAP to the magnitude of the shear rate therefore only depends on how many neurons fire and to what degree they are synchronized. The model predicts that the highest acceleration stimulus (~2.6 mg) evoked action potentials with a mean latency of ~0.7 ms relative to the stimulus onset, while the lowest acceleration stimulus (~0.7 mg) evoked action potentials with a mean latency of ~1.5 ms relative to the stimulus onset (Figures 5A,D). The action potential latency arises in the model from the integration time required for input current to depolarize the afferent neuron to threshold, not from synaptic delay. Convolving the action potentials with the extracellular field potential kernel (Eq. 12) provides the simulated vCAP in Figure 5E. Stimulus dependent latency of the simulated vCAP compares favorably to the measured vCAP (Figure 5F). Although there are differences in the detailed shape of the simulated vCAP relative to the experimental data (Figures 5E,F), the latency to peak is quite close to the data over all magnitudes simulated. Driving action potential generation with shear ϑ instead of shear rate $\dot{\vartheta }$ (i.e., $g_{1}\;vs.g_{2}$ in Eq. 9) extends the latency for the lowest strength stimulus well beyond that found in the data (compare ∆_R_ vs. ∆_S_ in Figure 6), suggesting phase-locked action potentials are driven by the bundle shear rate rather than bundle shear. The putative origin of shear rate sensitivity is addressed in the Discussion.

### Air conducted sound: Theory versus experiment

**Figure 7:**
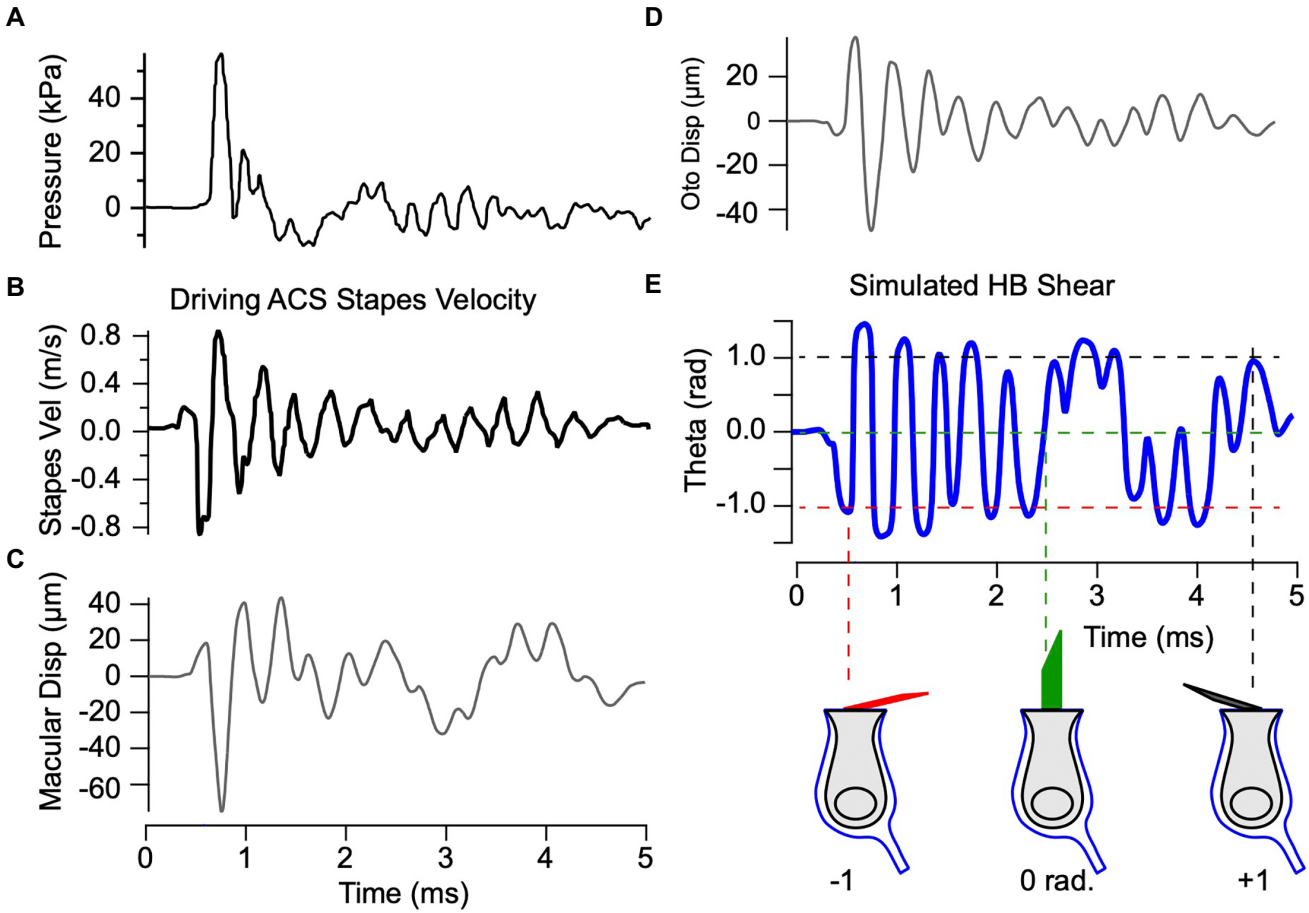
Predicted mechanical response to ACS blast waves. Model parameters are the same as previous figures. **(A,B)** Blast induced pressure and stapes velocity based on measurements from Jiang et al. (32). **(C,D)** Simulated displacement of the epithelial and otoconial layers in response to the experimentally measured stapes velocity in panel **B**. **(E)** Simulated shear acting to deflect hair bundles, predicting saturating deflections of hair bundles that would be expected to immediately destroy physiological function.

To determine if the same model applies to ACS we simulated vCAPs in response to brief pulses at 77, 81, 83, 86, 89, 92, 94, and 96 dB SPL (Figure 7) using exactly the same parameters as BCV (Figures 3–5). The input to the model was the LDV measured stapes velocity, shown as a series of curves in Figure 7A. The model predicts the simulated hair bundle shear shown in Figure 7B and shear rate shown in Figure 7C. The magnitude of hair bundle shear in response to ACS is predicted to be almost identical to BCV, but the waveform and frequency content differs considerably. The bundle shear rate (Figure 7C) was used to drive the simulation of action potential generation and vCAPs, again using exactly the same parameters as Figure 5 for BCV. The simulated vCAP (Figure 7E) compares remarkably well to the experimentally measured vCAP in magnitude, latency and waveform, especially given the fact that key parameters were estimated from the BCV data, not ACS data. The only parameter estimated from ACS data was α , which only scales the magnitude of the vCAP and does not change the temporal waveform. Consistent with simulations for BCV, there was no delay in the mechanical response from the onset of stapes velocity, but there was a stimulus level dependent latency between the stimulus onset and mean action potential firing time ranging from ~0.41 ms for the highest stapes velocity (~130 μm/s) to 0.74 ms for the lowest stapes velocity (~10 μm/s). Shorter latencies in response to stapes vibration vs. BCV are consistent with the experimental data, and were predicted by the model without changing any parameters. The latency is shorter for ACS vs. BCV in the model because the magnitude of the hair bundle shear rate was predicted to be higher (c.f. Figures 5C, 7C).

### Simulated blast exposure

**Figure 8:**
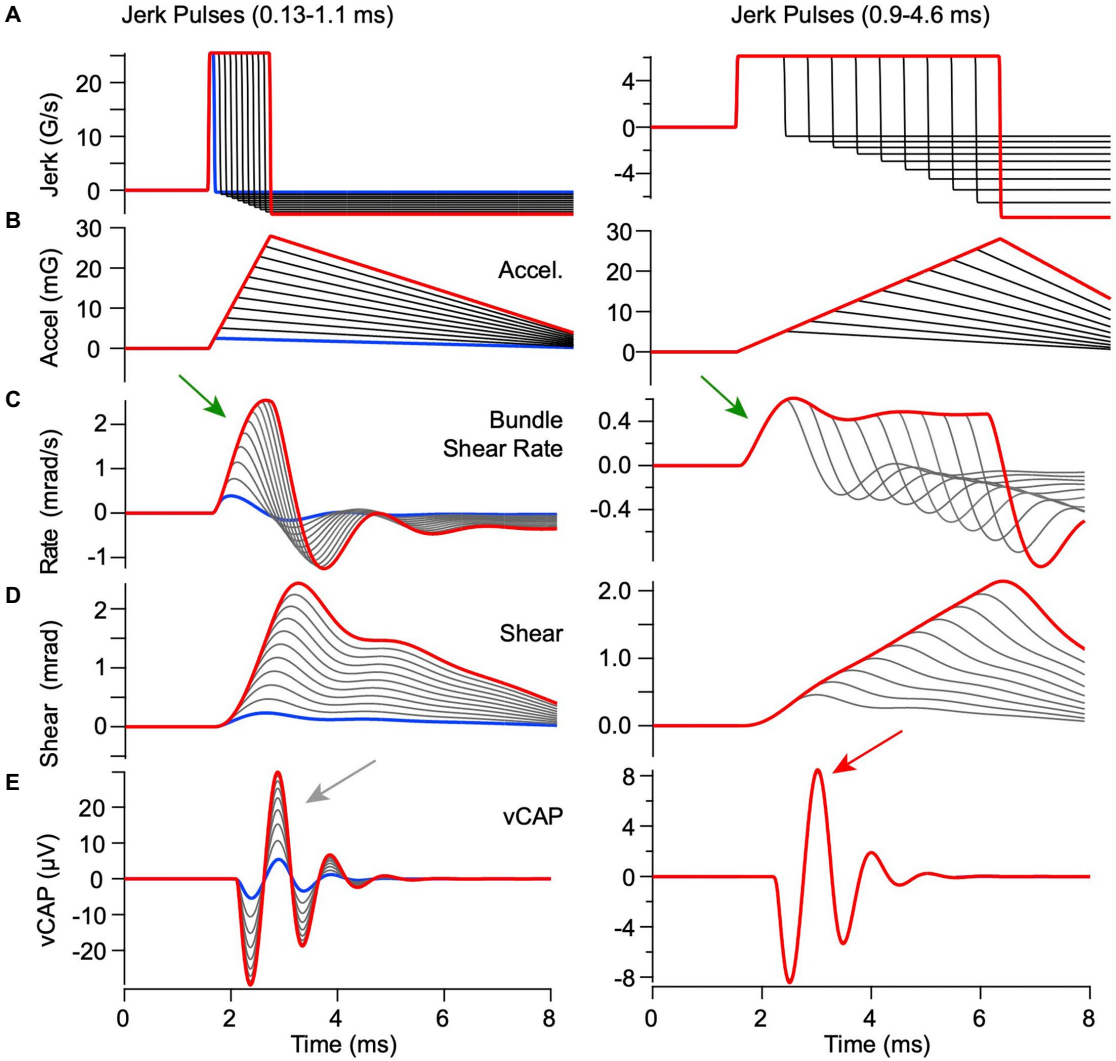
Predicted responses to BCV stimuli with constant peak linear jerk but variable acceleration. Model parameters are the same as previous figures. **(A)** linear jerk, **(B)** linear acceleration, **(C)** hair bundle shear rate, **(D)** hair bundle shear, and **(E)** vCAP. Constant jerk stimuli with short durations <0.8 ms [**(A)** left] evoked hair bundle shear [**(D)** left] and shear rate [**(C)** left] that increased in proportion to peak acceleration [**(B)** left] thus leading to vCAPs that increased magnitude with pulse width [**(E)** left, curves scale with acceleration, arrow]. In contrast, constant jerk stimuli with long durations >0.9 ms [**(A)** right] all evoked the same bundle shear rate within the first 1 ms of the stimulus [**(C)** right, arrow] irrespective of the peak acceleration, thus triggering equivalent vCAPs [**(E)** right, all curves superimposed, arrow]. Results demonstrate a switch from acceleration as the adequate stimulus for short pulses to jerk as the adequate stimulus for long pulses.

Having validated the mechanical model based on measured vibrations of the macula (Figure 3) in response to BCV and ACS (Figures 5, 7), we applied the model to examine potential mechanical damage to the utricle caused by exposure to loud ACS blast waves. We used the exact same parameters extracted from guinea pig data and drove the model with stapes velocity in response to blast exposure based on human cadaver data from Jiang et al. (32) (Figures 8A,B). Simulations were carried out for both ACS and BCV waveforms (32), and demonstrated that the response was dominated by stapes vibration rather than BCV (not shown). Displacement of the epithelial and otoconial layers in response to blast evoked stapes motion are predicted to exceed 40 μm relative to the temporal bone (Figures 8C,D) – a prediction orders of magnitude higher than measured for physiological levels of BCV and ACS. The model predicts that hair bundles experience shear exceeding ± 1 radian in response to blast exposure, rapidly displacing from upright to lying almost flat on the epithelial surface within ~150 μs of the initial stapes motion. The mechanical model is linear and does not include a mechanical failure mechanism, but if these estimates are correct one would expect the utricular hair bundles to be severely damaged by acute blast exposure during the first cycle, likely followed by hair cell death.

### Sensitivity to linear jerk and acceleration

In previous reports, VsEP magnitude increased in proportion to linear jerk (rate of change of acceleration, G/s) over a broad range of linear accelerations tested (33), a correlation that was not uniformly present in the guinea pig interaural vCAPs analyzed here (16). To determine the origin of this important difference, we simulated utricular responses for constant jerk pulses ranging from 0.13–4.6 ms in length, while allowing the acceleration to vary, and simulated utricular responses to constant acceleration pulses while allowing the jerk to vary. For the constant magnitude linear jerk stimuli (Figure 6), there were dramatic changes in hair bundle shear and shear rate waveforms with jerk pulse-width duration. The differences with jerk pulse width arise directly from the low-pass character of the mechanical response. Peak shear rate is the key mechanical variable driving vCAP responses. It’s important to note that peak shear rate increased in proportion to acceleration for short duration jerk pulse widths (<0.8 ms, Figure 6C left, arrow), because the peak hair bundle shear rate takes time to build up. In contrast, peak shear rate was constant for long duration jerk pulse widths (>9 ms, Figure 6C right, arrow). As a consequence, the vCAP magnitude scaled in proportion to temporal bone acceleration for short duration jerk pulses (Figure 6E left gray arrow, traces dispersed), but scaled in proportion to linear jerk for long duration jerk pulses (Figure 6E right red arrow, all traces superimposed). Note, the magnitude of the short duration jerk stimuli (Figure 6, left) is larger than the long duration (Figure 6, right) to match the range of acceleration (Figure 6B, left vs. right), thus resulting in a smaller vCap response for long duration stimuli (Figure 6E, left vs. right).

**Figure 9:**
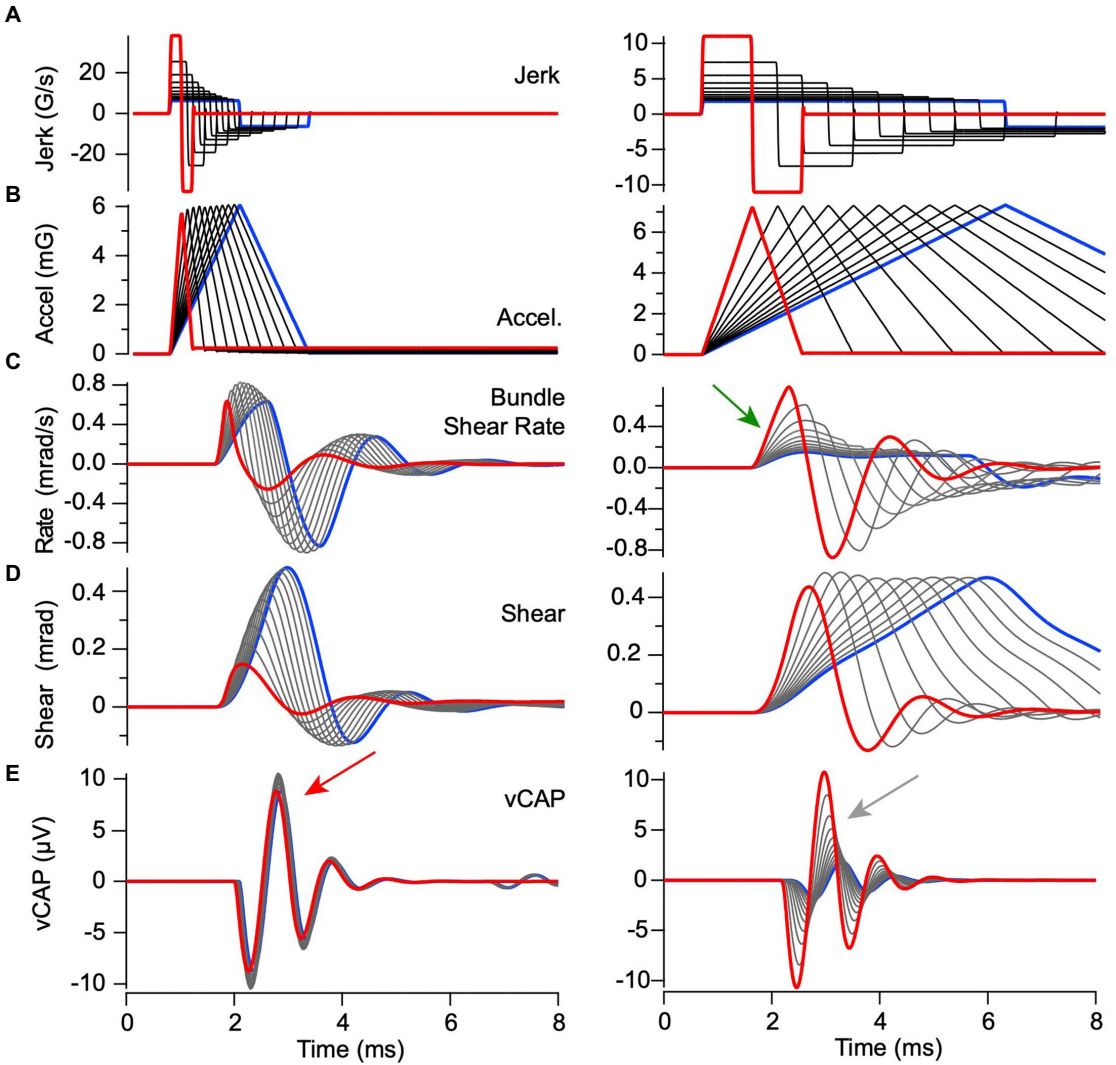
Predicted responses to BCV stimuli with constant peak acceleration but variable linear jerk. Model parameters are the same as previous figures and format of **(A–E)** is the same as Figure 8. Acceleration stimuli with rise times <0.8 ms evoked nearly equivalent onset bundle shear rates [**(C)** left] and vCAPs [**(E)** left], but stimuli with rise times >0.8 ms evoked bundle shear rates [**(C)** left] and vCAPs [(Figure 6E) right] that scaled with linear acceleration. Results demonstrate a shift from acceleration sensitivity for high frequency stimuli to jerk sensitivity for low frequency stimuli, with a corner frequency near 1 kHz in adult guinea pigs.

The mechanical part of the model is linear, so the same pulse width conclusion was found when driving the model with pulses of linear acceleration vs. pulses of linear jerk (Figures 6, 9). Results for constant peak linear acceleration are provided in Figure 9, showing jerk, acceleration, bundle shear rate, bundle shear and vCAP in rows A-E, respectively. Consistent with Figure 6C, peak shear rate is the key mechanical variable driving vCAP responses (Figure 9C). Short acceleration pulses with rise times <0.8 ms had variable peak jerk, but all evoked similar same peak shear rates (Figure 9C left) and almost identidcal vCAPs (Figure 9E left, red arrow). In contrast, long acceleration pulses with rise times >0.9 ms evoked variable peak shear rates (Figure 9C right) and vCAPs that scaled with peak linear jerk (Figure 9E right, gray arrow).

The switch from acceleration sensitivity to jerk sensitivity with increasing pulse width can also be described in terms of frequency content of the stimulus. For the parameters in Table 1, the eigenvalues of Eq. 1–4 yield two natural frequencies of vibration: 495 Hz and 565 Hz, with each corresponding to different relative motion between the otoconial layer and the epithelium. As the BCV stimulus frequency is increased, the hair bundle shear rate driving synchronized action potentials shifts form a low frequency jerk sensitive mode to a high frequency acceleration sensitive mode. The transition in guinea pig occurs near 530 Hz, which corresponds to a full-cycle stimulus period of 1.8 ms and half-cycle positive pulse width of 0.9 ms, consistent with time-domain simulations in Figures 6, 9. When the jerk frequency content in the BCV or ACS stimuli is high, the vCAP responses scale more closely with linear acceleration rather than jerk (16).

## Discussion

vCAPs are extracellular voltages that arise from the synchronized firing of a large number of afferent neurons in response to transient inertial, vibrational or acoustic stimuli. The neurons most sensitive to transient stimuli make calyceal synaptic contacts with type I vestibular hair cells in the striolar region of the sensory epithelium (1, 34–36). The unique calyx synapse supports glutamatergic quantal transmission (37, 38) as well as two forms of nonquantal (NQ) transmission: an ultrafast nonquantal component (NQf) that operates through direct resistive coupling and a slow nonquantal component (NQs) involving K^+^ build up in the synaptic cleft (22–25). The ultrafast resistive NQf allows the modulated MET current to almost immediately alter hair cell and postsynaptic voltage without delay, giving rise to short-latency vCAPs that persist even after blocking quantal transmission pharmacologically (39, 40). The present model describing generation of short latency synchronized action potentials therefore assumed depolarizing current entered the calyx terminal without delay (Eq. 9). The model also ignored regularly firing neurons (by setting $g_{0}=0,g_{1}=0$ ) because they do not synchronize action potential firing times to transient stimuli. An absolute refractory period was included, limiting the short latency responses reported here to a single action potential for each responding neuron, which combine to give synchronized action potential times shown as post stimulus histograms in Figures 5D, 7D (Eq. 11). The population of action potential times determines the timing and magnitude of the extracellular vCAP through a linear convolution with a voltage kernel (Eq. 12). As a result, the waveform of the vCAP was a summation of multiple kernel waveforms, each shifted in time to reflect spike timing and amplified to reflect the number of neurons recruited at each time. The kernel waveform used here is empirical and based on features of vCAPs recorded adjacent to the nerve in guinea pig (16), but is quite similar to the waveform in auditory nerve CAPs (29). Applying the present model to VsEPs recorded using subcutaneous electrodes (41) would require a different kernel accounting for the conduction pathway from the nerve to the recording sites, but the mechanical model and spike generation properties would be expected to translate to alternative experimental conditions providing parameters are appropriately selected for the species and specific stimulus used.

From a biomechanical perspective, hair bundle shear-rate was the organ level mechanical variable that correlated with the magnitude and timing of vCAPs evoked by BCV (Figure 5) and ACS (Figure 7). This means action potential generation increased in proportion to hair bundle velocity rather than displacement for both stimuli. Previous results demonstrate afferent neurons responding to ACS and BCV are the same type, have irregular discharge properties and innervate the striola (42–44). Evidence from mammalian type I hair cells and calyx bearing afferent neurons suggests a majority of the signal processing responsible for velocity sensitivity and synchronization occurs postsynaptically (28). A postsynaptic origin is consistent with whole-organ microphonics in the guinea pig utricle, which reflects the phase of the MET current and is nearly in phase with hair bundle displacement (16, 18). Similar results were reported in the toadfish crista where step hair bundle displacements evoked rapidly adapting afferent discharge in units sensitive to high frequencies, while microphonics and hair cell receptor potentials closely followed the step displacement of the bundle (27, 45–47). Together, available data support the hypothesis that vestibular hair cell depolarization primarily reflects hair bundle shear (displacement), while spike generation in the most highly synchronized afferent neurons primarily reflects hair bundle shear rate, with rate sensitivity arising from signal processing interposed between the MET current and spike timing.

The present mechanical model was also applied to estimate utricular hair bundle deflection caused by blast exposure. It has been shown in rodents that a bilateral 63 kPa (~190 dB) blast results in eardrum perforations and likely permanent loss of stereocilia in the utricle (48), and a 137 kPa (~197 dB) unilateral blast reduces spontaneous discharge and sensitivity of afferents with regular and irregular inter-spike intervals without significant acute loss of steady state VOR (49, 50). Humans experience persistent vestibular symptoms and increased incidence of BPPV following blast exposure (51), consistent with the hypothesis that the otolith organs are uniquely vulnerable to blast injury (52), putatively due to the close proximity of the utricle to the stapes (53). The present mechanical model was validated using direct LDV measurements of macular vibration in response to stapes vibration, and hence provides a means to estimate utricular mechanics for any prescribed stapes motion, including blast. Simulations summarized in Figure 8 are rough approximations, in part because the stapes velocity used was based on measurements from human cadavers (32). Also, the mechanical model is linear, which would be only a crude approximation given the magnitude of blast forces. Nevertheless, results predict large angular deflection of hair bundles consistent with the hypothesis that blast-level ACS leads to immediate utricular damage through extensive hair bundle shear at a level that would be expected to disrupt the otoconia and damage hair cells. Individuals suffering from conditions that compromise integrity of the utricle may be particularly vulnerable to head impact and loud sounds (54–56), and combining the model with additional experimental data has potential to improve our understanding of trauma-induced otolith dysfunction in healthy and diseased populations.

Present results (Figure 6) are consistent with previous reports in aves and rodents that linear jerk is the adequate stimulus to evoke vCAPs (or VsEPs) for relatively long duration stimuli (33, 41, 57), but also reveal a switch in the adequate stimulus to linear acceleration for shorter duration jerk stimuli. In the guinea pig, the switch occurs for jerk pulse widths near ~0.8 ms, shorter jerk pulse widths evoke vCAPs that scaled with linear acceleration of the temporal bone and longer pulse widths evoked vCAPs scaled with linear jerk. The frequency characterizing the switch from acceleration jerk sensitivity depends on the mechanics and would be higher in rodents with a smaller utricle, but lower for larger mammals including humans. Caution therefore should be exercised when extending findings for high frequency BCV and ACS from small rodents to humans. Given human utricular morphology and the inertia of the head, clinically relevant short-duration stimuli (e.g., mechanical tap (58, 59)) likely evoke synchronized neural responses that scale with linear acceleration rather than jerk (Figure 9, left).

Finally, it is important to recognize the mechanical part of the model is simplified to 2-DOF, and the neuronal part of the model is strictly empirical. The mechanical part of the model is linearized and assumes vibration moves along a straight line – assumptions that are only crude approximations and clearly cannot address 3-D vibration or directly predict damage during blast. The neural model lumps the hair cell, calyx and afferent into one highly simplified empirical IAF model driven by fast NQ transmission, ignoring specific ion channels and other key biophysical factors including quantal synaptic delay and slow K^+^ build up in the synaptic cleft. The vCAP unitary waveform kernel also was selected empirically based on the experimentally measured waveform period and decay time constant. Implementing a more realistic biophysical model of hair cell responses and action potential generation will be an important step to understand the specific origin(s) of shear rate sensitivity. We should also point out that the present model is for the guinea pig subject to relatively low levels of intra-aural BCV vibration and ACS. Including additional forms of synaptic transmission and recruitment of less sensitive fibers would be required to extend the neural part of the model to address more intense vibrational stimuli commonly used for VsEP phenotyping in small rodents (41). For the present model to reproduce VsEP waveforms reported previously would also require a different extracellular voltage kernel (Eq. 12) to account for differences in the extracellular recording sites, and would require adjustment of mechanical parameters to account for differences in morphology and properties between species.

## Conclusion

The present study was designed to understand the biomechanical and neuronal mechanisms responsible for synchronization of utricular afferent firing in response to transient stimuli commonly used in the clinic (VEMPs) and in the laboratory (VsEP). A highly simplified mathematical model was developed and validated by direct comparison to measured macular velocity and vCAPs in response to brief (0.5–3 ms) packets of vibration. Unlike previous models of transduction by the utricle, the present analysis includes vibration of the membranous labyrinth relative to the temporal bone, inertial coupling to the stapes, hair bundle shear, action potential generation and vCAP simulation. Results demonstrate the key mechanical variable driving synchronized action potential generation is the hair bundle shear rate (rad-s^−1^). Using a single parameter set, results reproduce macular velocity and vCAPs to both BCV and ACS with surprising fidelity, and suggest the model can be used to design species-specific stimuli to achieve controllable hair bundle shear and synchronized neural responses to meet the specific clinical or scientific goals.

## Data availability statement

The original contributions presented in the study are included in the article/supplementary material, further inquiries can be directed to the corresponding author/s.

## Ethics statement

The animal study was reviewed and approved by University of Sydney Animal Ethics Committee.

## Author contributions

CP, DB, and IC designed the experiments. CP performed the experiments. RR and SJ conceptualized the CP model. HZ and WZ conceptualized the blast simulations. RR formulated the mathematical models, wrote the simulation code, and wrote the original manuscript. RR and NG ran the simulations. All authors contributed to the article and approved the submitted version.

## Funding

This work was supported by NIH DC018919 (HZ) and NIH DC 006685 (RR) and a Macquarie University Research Fellowship (MQRF) (CP).

## Conflict of interest

The authors declare that the research was conducted in the absence of any commercial or financial relationships that could be construed as a potential conflict of interest.

## Publisher’s note

All claims expressed in this article are solely those of the authors and do not necessarily represent those of their affiliated organizations, or those of the publisher, the editors and the reviewers. Any product that may be evaluated in this article, or claim that may be made by its manufacturer, is not guaranteed or endorsed by the publisher.

## References

[1] Curthoys IS Burgess AM Goonetilleke SC. Phase-locking of irregular Guinea pig primary vestibular afferents to high frequency (>250Hz) sound and vibration. Hear Res. (2019) 373:59–70. doi: 10.1016/j.heares.2018.12.009, PMID: 30599427

[2] Huang J Tang X Xu Y Zhang C Chen T Yu Y. Differential activation of canal and otolith afferents by acoustic tone bursts in rats. J Assoc Res Otolaryngol. (2022) 23:435–53. doi: 10.1007/s10162-022-00839-1, PMID: 35378621 PMC9086073

[3] Young ED Fernandez C Goldberg JM. Responses of squirrel monkey vestibular neurons to audio-frequency sound and head vibration. Acta Otolaryngol. (1977) 84:352–60. doi: 10.3109/00016487709123977, PMID: 303426

[4] Erlanger J Gasser H. The compound nature of the action current of nerve as disclosed by the cathode ray oscillograph. Am J Phys. (1924) 70:624–66. doi: 10.1152/ajplegacy.1924.70.3.624

[5] Jones SM Erway LC Johnson KR Yu H Jones TA. Gravity receptor function in mice with graded otoconial deficiencies. Hear Res. (2004) 191:34–40. doi: 10.1016/j.heares.2004.01.008, PMID: 15109702

[6] Lee C Jones TA. Effects of several therapeutic agents on mammalian vestibular function: meclizine, diazepam, and JNJ7777120. J Assoc Res Otolaryngol. (2021) 22:527–49. doi: 10.1007/s10162-021-00803-5, PMID: 34009490 PMC8476680

[7] Goldberg JM. The Vestibular System: A Sixth Sense. New York: Oxford University Press (2012).

[8] Colebatch JG Rosengren SM Welgampola MS. Vestibular-evoked myogenic potentials. Handb Clin Neurol. (2016) 137:133–55. doi: 10.1016/B978-0-444-63437-5.00010-8 27638068

[9] Curthoys IS Grant JW Pastras CJ Brown DJ Burgess AM Brichta AM. A review of mechanical and synaptic processes in otolith transduction of sound and vibration for clinical VEMP testing. J Neurophysiol. (2019) 122:259–76. doi: 10.1152/jn.00031.2019, PMID: 31042414

[10] Rosengren SM Colebatch JG Young AS Govender S Welgampola MS. Vestibular evoked myogenic potentials in practice: methods, pitfalls and clinical applications. Clin Neurophysiol Pract. (2019) 4:47–68. doi: 10.1016/j.cnp.2019.01.005, PMID: 30949613 PMC6430081

[11] Taylor RL Welgampola MS Nham B Rosengren SM. Vestibular-evoked myogenic potential testing in vestibular localization and diagnosis. Semin Neurol. (2020) 40:18–32. doi: 10.1055/s-0039-3402068, PMID: 31935772

[12] Grant JW Cotton JR. A model for otolith dynamic response with a viscoelastic gel layer. J Vestib Res. (1990) 1:139–51. doi: 10.3233/VES-1991-1205, PMID: 1670147

[13] Rabbitt R Damiano E Grant J. Biomechanics of the vestibular semicircular canals and otolith organs In: Highstein SM Popper A Fay R, editors. The Vestibular System. NY: Springer Verlag (2003)

[14] Dunlap MD Grant JW. Experimental measurement of utricle system dynamic response to inertial stimulus. J Assoc Res Otolaryngol. (2014) 15:511–28. doi: 10.1007/s10162-014-0456-x, PMID: 24845403 PMC4141440

[15] Jones TA Lee C Gaines GC Grant JW. On the high frequency transfer of mechanical stimuli from the surface of the head to the macular neuroepithelium of the mouse. J Assoc Res Otolaryngol. (2015) 16:189–204. doi: 10.1007/s10162-014-0501-9, PMID: 25648882 PMC4368654

[16] Pastras C Curthoys I Rabbitt R Brown D. Vestibular compound action potentials and macular velocity evoked by sound and vibration in the Guinea pig. bioRxiv. (2022):510190. doi: 10.1101/2022.09.29.510190

[17] Pastras CJ Curthoys IS Brown DJ. Dynamic response to sound and vibration of the Guinea pig utricular macula, measured in vivo using laser Doppler Vibrometry. Hear Res. (2018) 370:232–7. doi: 10.1016/j.heares.2018.08.005, PMID: 30170855

[18] Pastras CJ Stefani SP Camp AJ Curthoys IS Brown DJ. Summating potentials from the utricular macula of anaesthetized Guinea pigs. Hear Res. (2021) 406:108259. doi: 10.1016/j.heares.2021.108259, PMID: 34038828

[19] Cooper NP Rhode WS. Basilar membrane tonotopicity in the hook region of the cat cochlea. Hear Res. (1992) 63:191–6. doi: 10.1016/0378-5955(92)90084-Z, PMID: 1464570

[20] Grant W Best W. Otolith-organ mechanics: lumped parameter model and dynamic response. Aviat Space Environ Med. (1987) 58:970–6. PMID: 3314853

[21] Nam JH Grant JW Rowe MH Peterson EH. Multiscale modeling of mechanotransduction in the utricle. J Neurophysiol. (2019) 122:132–50. doi: 10.1152/jn.00068.2019, PMID: 30995138 PMC6689775

[22] Contini D Holstein GR Art JJ. Synaptic cleft microenvironment influences potassium permeation and synaptic transmission in hair cells surrounded by calyx afferents in the turtle. J Physiol. (2020) 598:853–89. doi: 10.1113/JP278680, PMID: 31623011 PMC7024053

[23] Contini D Price SD Art JJ. Accumulation of K(+) in the synaptic cleft modulates activity by influencing both vestibular hair cell and calyx afferent in the turtle. J Physiol. (2017) 595:777–803. doi: 10.1113/JP273060, PMID: 27633787 PMC5285615

[24] Holt JC Chatlani S Lysakowski A Goldberg JM. Quantal and nonquantal transmission in calyx-bearing fibers of the turtle posterior crista. J Neurophysiol. (2007) 98:1083–101. doi: 10.1152/jn.00332.2007, PMID: 17596419 PMC3397384

[25] Lim R Kindig AE Donne SW Callister RJ Brichta AM. Potassium accumulation between type I hair cells and calyx terminals in mouse crista. Exp Brain Res. (2011) 210:607–21. doi: 10.1007/s00221-011-2592-4, PMID: 21350807

[26] Govindaraju AC Quraishi IH Lysakowski A Eatock RA Raphael RM. Nonquantal transmission at the vestibular hair cell-calyx synapse: K(LV) currents modulate fast electrical and slow K(+) potentials. Proc Natl Acad Sci U S A. (2023) 120:e2207466120. doi: 10.1073/pnas.2207466120, PMID: 36595693 PMC9926171

[27] Rabbitt RD Boyle R Holstein GR Highstein SM. Hair-cell versus afferent adaptation in the semicircular canals. J Neurophysiol. (2005) 93:424–36. doi: 10.1152/jn.00426.2004, PMID: 15306633 PMC3000937

[28] Songer JE Eatock RA. Tuning and timing in mammalian type I hair cells and calyceal synapses. J Neurosci. (2013) 33:3706–24. doi: 10.1523/JNEUROSCI.4067-12.2013, PMID: 23426697 PMC3857958

[29] Chertoff M Lichtenhan J Willis M. Click- and chirp-evoked human compound action potentials. J Acoust Soc Am. (2010) 127:2992–6. doi: 10.1121/1.3372756, PMID: 21117748 PMC3188627

[30] Goldstein M Kiang N. Synchrony of neural activity in electric responses evoked by transient acoustic stimuli. J Acoust Soc Am. (1958) 30:107–14. doi: 10.1121/1.1909497

[31] Goldberg JM Brown PB. Response of binaural neurons of dog superior olivary complex to dichotic tonal stimuli: some physiological mechanisms of sound localization. J Neurophysiol. (1969) 32:613–36. doi: 10.1152/jn.1969.32.4.613, PMID: 5810617

[32] Jiang S Dai C Gan RZ. Dual-laser measurement of human stapes footplate motion under blast exposure. Hear Res. (2021) 403:108177. doi: 10.1016/j.heares.2021.108177, PMID: 33524791

[33] Jones TA Jones SM Vijayakumar S Brugeaud A Bothwell M Chabbert C. The adequate stimulus for mammalian linear vestibular evoked potentials (VsEPs). Hear Res. (2011) 280:133–40. doi: 10.1016/j.heares.2011.05.005, PMID: 21664446 PMC3826178

[34] Desai SS Zeh C Lysakowski A. Comparative morphology of rodent vestibular periphery. I saccular and utricular maculae. J Neurophysiol. (2005) 93:251–66. doi: 10.1152/jn.00746.2003, PMID: 15240767 PMC12456082

[35] Goldberg JM Desmadryl G Baird RA Fernandez C. The vestibular nerve of the chinchilla. IV. Discharge properties of utricular afferents. J Neurophysiol. (1990) 63:781–90. doi: 10.1152/jn.1990.63.4.781, PMID: 2341876

[36] Zhu H Tang X Wei W Maklad A Mustain W Rabbitt R. Input-output functions of vestibular afferent responses to air-conducted clicks in rats. J Assoc Res Otolaryngol. (2014) 15:73–86. doi: 10.1007/s10162-013-0428-6, PMID: 24297262 PMC3901862

[37] Highstein SM Mann MA Holstein GR Rabbitt RD. The quantal component of synaptic transmission from sensory hair cells to the vestibular calyx. J Neurophysiol. (2015) 113:3827–35. doi: 10.1152/jn.00055.2015, PMID: 25878150 PMC4473518

[38] Rennie KJ Streeter MA. Voltage-dependent currents in isolated vestibular afferent calyx terminals. J Neurophysiol. (2006) 95:26–32. doi: 10.1152/jn.00641.2005, PMID: 16162827

[39] Irons-Brown SR Jones SM Jones TA. The simultaneous in vivo perilymphatic perfusion of avian auditory and vestibular end organs. J Neurosci Methods. (2003) 131:57–64. doi: 10.1016/S0165-0270(03)00239-5, PMID: 14659824

[40] Irons-Brown SR Jones TA. Effects of selected pharmacological agents on avian auditory and vestibular compound action potentials. Hear Res. (2004) 195:54–66. doi: 10.1016/j.heares.2004.02.011, PMID: 15350279

[41] Jones TA Jones SM. Short latency compound action potentials from mammalian gravity receptor organs. Hear Res. (1999) 136:75–85. doi: 10.1016/S0378-5955(99)00110-0, PMID: 10511626

[42] Curthoys IS Vulovic V. Vestibular primary afferent responses to sound and vibration in the Guinea pig. Exp Brain Res. (2011) 210:347–52. doi: 10.1007/s00221-010-2499-5, PMID: 21113779

[43] Curthoys IS Vulovic V Burgess AM Sokolic L Goonetilleke SC. The response of Guinea pig primary utricular and saccular irregular neurons to bone-conducted vibration (BCV) and air-conducted sound (ACS). Hear Res. (2016) 331:131–43. doi: 10.1016/j.heares.2015.10.019, PMID: 26626360

[44] Curthoys IS Vulovic V Sokolic L Pogson J Burgess AM. Irregular primary otolith afferents from the Guinea pig utricular and saccular maculae respond to both bone conducted vibration and to air conducted sound. Brain Res Bull. (2012) 89:16–21. doi: 10.1016/j.brainresbull.2012.07.007, PMID: 22814095

[45] Iversen MM Christensen DA Parker DL Holman HA Chen J Frerck MJ. Low-intensity ultrasound activates vestibular otolith organs through acoustic radiation force. J Acoust Soc Am. (2017) 141:4209–19. doi: 10.1121/1.4984287, PMID: 28618821 PMC5552392

[46] Rabbitt RD Breneman KD King C Yamauchi AM Boyle R Highstein SM. Dynamic displacement of normal and detached semicircular canal cupula. J Assoc Res Otolaryngol. (2009) 10:497–509. doi: 10.1007/s10162-009-0174-y, PMID: 19513793 PMC2774407

[47] Rabbitt RD Highstein SM Boyle R. Determinants of semicircular canal afferent response dynamics in fish. Ann N Y Acad Sci. (1996) 781:213–43. doi: 10.1111/j.1749-6632.1996.tb15703.x, PMID: 8694417

[48] Lien S Dickman JD. Vestibular injury after low-intensity blast exposure. Front Neurol. (2018) 9:297. doi: 10.3389/fneur.2018.00297, PMID: 29867715 PMC5960675

[49] Sandlin DS Yu Y Huang J Zhang C Arteaga AA Lippincott JK. Autonomic responses to blast overpressure can be elicited by exclusively exposing the ear in rats. J Otol. (2018) 13:44–53. doi: 10.1016/j.joto.2018.01.001, PMID: 30559764 PMC6291641

[50] Yu Y Huang J Tang X Allison J Sandlin D Ding D. Exposure to blast shock waves via the ear canal induces deficits in vestibular afferent function in rats. J Otol. (2020) 15:77–85. doi: 10.1016/j.joto.2020.01.003, PMID: 32884557 PMC7451608

[51] Akin FW Murnane OD Hall CD Riska KM. Vestibular consequences of mild traumatic brain injury and blast exposure: a review. Brain Inj. (2017) 31:1188–94. doi: 10.1080/02699052.2017.1288928, PMID: 28981340

[52] Akin FW Murnane OD. Head injury and blast exposure: vestibular consequences. Otolaryngol Clin N Am. (2011) 44:323–334, viii. doi: 10.1016/j.otc.2011.01.005, PMID: 21474007

[53] Mukherjee P Uzun-Coruhlu H Curthoys IS Jones AS Bradshaw AP Pohl DV. Three-dimensional analysis of the vestibular end organs in relation to the stapes footplate and piston placement. Otol Neurotol. (2011) 32:367–72. doi: 10.1097/MAO.0b013e3182096ddd, PMID: 21283036

[54] Roman-Naranjo P Gallego-Martinez A Soto-Varela A Aran I Moleon MDC Espinosa-Sanchez JM. Burden of rare variants in the OTOG gene in familial Meniere's disease. Ear Hear. (2020) 41:1598–605. doi: 10.1097/AUD.0000000000000878, PMID: 33136635

[55] Roman-Naranjo P Parra-Perez AM Escalera-Balsera A Soto-Varela A Gallego-Martinez A Aran I. Defective alpha-tectorin may involve tectorial membrane in familial Meniere disease. Clin Transl Med. (2022) 12:e829. doi: 10.1002/ctm2.829 35653455 PMC9162437

[56] Senofsky N Faber J Bozovic D. Vestibular drop attacks and Meniere's disease as results of Otolithic membrane damage-a numerical model. J Assoc Res Otolaryngol. (2022) 24:107–15. doi: 10.1007/s10162-022-00880-0, PMID: 36517730 PMC9971529

[57] Jones SM Jones TA. Short latency vestibular evoked potentials in the chicken embryo. J Vestib Res. (1996) 6:71–83. doi: 10.3233/VES-1996-6202, PMID: 8925118

[58] Macdougall HG Holden J Rosengren SM Chiarovano E. muVEMP: a portable Interface to record vestibular evoked myogenic potentials (VEMPs) with a smart phone or tablet. Front Neurol. (2018) 9:543. doi: 10.3389/fneur.2018.00543, PMID: 30026727 PMC6042498

[59] Rodriguez AI Marler E Fitzpatrick D Creutz T Cannon SA Thomas MLA. Optimization of cervical and ocular vestibular evoked myogenic potential testing using an impulse hammer in adults, adolescents, and children. Otol Neurotol. (2020) 41:817–27. doi: 10.1097/MAO.0000000000002632, PMID: 32221109 PMC7311239

